# Contribution of an integrative approach to the oral rehabilitation of a bipolar patient

**DOI:** 10.1192/j.eurpsy.2023.1466

**Published:** 2023-07-19

**Authors:** J. D. Bidgoli

**Affiliations:** Dentistry, Cliniques Universitaires Saint-Luc (UCLOUVAIN), Brussels, Belgium

## Abstract

**Introduction:**

Ironically, the correlation between systemic pathologies and caries/ periodontal diseases is commonly accepted by the scientific and medical community, but the fact that severe mental illnesses may affect one’s physical health, and thus lead to poor oral health is less well-known.

**Objectives:**

This clinical case report’s aim is to raise awareness among medical staff about the relevance of appropriate management of patients with severe mental illnesses in terms of dental care.

**Methods:**

Illustration of integrative management through the description of the oral rehabilitation of a 63-year-old female whose bipolar disorder had been diagnosed 20 years ago. This patient came in May 2020 to the Cliniques Universitaires Saint-Luc (Brussels, Belgium) for painless mobility of the lower central incisors. Her diagnosis later revealed more than 20 advanced carious lesions as well as chronic severe periodontitis. Since then, despite numerous dental treatments and regular follow-up appointments, an important degradation of her oral health could be noted over time, characterized by a recurrent carious phenomenon (called “rampant” caries) and a failure in the stabilization of her periodontitis.

**Results:**

The literature review revealed that bipolarity was a major risk factor leading to tooth decay and aggravation of periodontal disease; when bipolar disorders’ inherent symptoms are coupled with medication’s side effects, they work in synergy towards a deterioration of oral health. Following the results obtained after this etiological research, the management of this patient was pursued in accordance with specific recommendations proposed in the literature. A stabilization of her oral condition was then observed at the 3-6-9-12 month check-ups, without the appearance of any new lesion.

**Image:**

**Figure:**
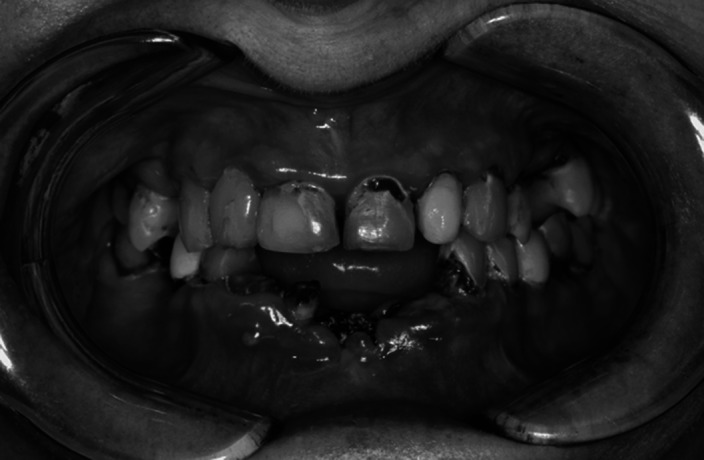

**Image 2:**

**Figure:**
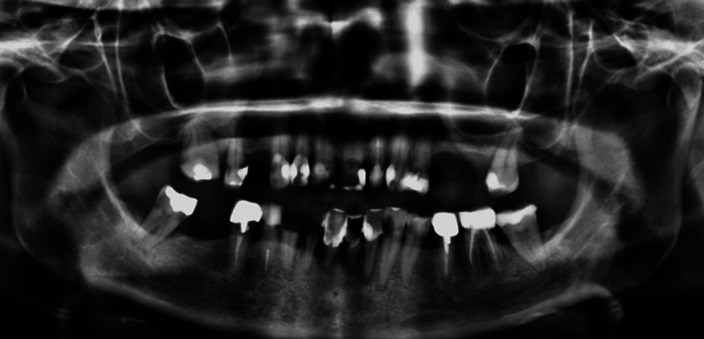

**Conclusions:**

The positive effect obtained on this patient’s oral health following the implementation of bipolar disorders specific measures opens the discussion on the relevance of integrating individual strategies within the therapeutic management of patients with severe mental illnesses.

**Disclosure of Interest:**

None Declared

